# Introduction and Testing of a Monitoring and Colony-Mapping Method for Waterbird Populations That Uses High-Speed and Ultra-Detailed Aerial Remote Sensing

**DOI:** 10.3390/s140712828

**Published:** 2014-07-18

**Authors:** Gábor Bakó, Márton Tolnai, Ádám Takács

**Affiliations:** 1 Interspect Research Group, Rákóczi Ferenc út 42, 2314 Halásztelek II, Hungary; E-Mails: tolnaimarton@interspect.hu (M.T.); takacsadam@interspect.hu (Á.T.); 2 Institute of Botany and Plant Ecophysiology, Szent István University, Páter K. u. 1., 2100 Gödöllő, Hungary

**Keywords:** waterbirds, biodiversity, population survey, environmental monitoring, aerial photogrammetry, terrain mapping

## Abstract

Remote sensing is a method that collects data of the Earth's surface without causing disturbances. Thus, it is worthwhile to use remote sensing methods to survey endangered ecosystems, as the studied species will behave naturally while undisturbed. The latest passive optical remote sensing solutions permit surveys from long distances. State-of-the-art highly sensitive sensor systems allow high spatial resolution image acquisition at high altitudes and at high flying speeds, even in low-visibility conditions. As the aerial imagery captured by an airplane covers the entire study area, all the animals present in that area can be recorded. A population assessment is conducted by visual interpretations of an ortho image map. The basic objective of this study is to determine whether small- and medium-sized bird species are recognizable in the ortho images by using high spatial resolution aerial cameras. The spatial resolution needed for identifying the bird species in the ortho image map was studied. The survey was adjusted to determine the number of birds in a colony at a given time.

## 1. Introduction

Today, aerial remote sensing surveys are becoming more popular as they can be used to monitor animals in their natural habitat without disturbing them. Previously, the details achievable by high spatial resolution aerial imagery enabled the population estimation of only a few bird species. Recently, high spatial resolution and high-speed digital aerial cameras have recorded open land surfaces at high altitudes during high-speed flights. Thus, animals do not perceive the aircraft passing over them, and due to the rapid survey, the movement of individual birds does not cause errors in the evaluation. Orthophoto maps are created by the aerial images in which individual birds of different species are identified. It is important to eliminate the possibility of double counting between flight lines of even the most mobile animals; thus, this risk is reduced by the use of high flying speeds and appropriate flight mission planning. In this way, the vegetation and the land cover of the habitat can be mapped, the number of individual birds of the colonies can be counted and the behavior of individual birds can be noted and analyzed.

## 2. Background

### 2.1. Previous Results of Aerial Animal Population Surveys

During observer-based aerial animal counting, the altitude and the flight speed influence the detection probability, and the accuracy varies [1]. The subjective perception of the observer significantly distorts the results [2]. Therefore, image recording methods come to the fore, which reduce the subjectivity involved and also decrease negative perceptions and bias. Coordinate-based positioning is an indispensable part of modern visual observations; satellite-based navigation systems (e.g., GPS) record the flight track of an airplane and the exact location of the observed area [3]. Coordinates are assigned to each pixel of the acquired orthophotos and georeferenced images, and these images can subsequently be re-analyzed by multiple interpreters. Spatial aspects of the information obtained are assured by the use of photogrammetry, direct georeferencing and further image processing methods [4].

Recently, numerous studies also discuss the use of micro unmanned aerial vehicle (UAV) technology in population estimations; UAVs can deliver fine spatial resolution data at temporal resolutions defined by the end user [5]. For wildlife counting based on aerial remote sensing, the recording speed is critical if large areas are to be surveyed over a short time. Therefore, the use of faster, fixed-wing aircraft is required. Even so, meeting the inter-related criteria of spatial detail, image quality, coverage, minimal animal disturbance, minimal animal movement (to avoid the risk of double counting), and safety has been impossible without making compromises.

At slower flights speeds (e.g., <170 km/h), the light conditions and, in some cases, even the test conditions change before the recording for the entire survey area can be completed. Previously, aerial animal counting at ground speeds of over 200 km/h was possible only in special cases, e.g., large-bodied mammals in the savanna or northern gannet (*Morus bassanus*) colonies.

These aerial surveys were successful because the habitats of these animals were limited and the size of the surveyed species was relatively large. The classical aerial survey in the case of large-bodied animals provides fast and accurate results, mainly in treeless habitats. For example, in the Tsavo-Mkozami West National Park, the population decline of elephants (*Loxodonta africana*) and buffalos (*Syncerus caffer*) was detected by these methods [6]. Similarly, the classical aerial surveys of polar bears (*Ursus maritimus*) were successful in Alaska [7]. In the case of northern gannets, their body size and their limited habitat has also enabled them to be observed. On Ramsey Island, the borders of gannet colonies have been surveyed since 1984. However, improved methods now allow researchers to count individual birds [8]. The survey was possible because colonies were seated on treeless islands and cliffs where breeding birds were concentrated in extended and well-demarcated areas. This enabled precise observations to be made. If the breeding colonies were recorded at a discrete time, then the movement of the birds was not a problem. In the cases of other species where the habitats are not as well demarcated, the classical methods can have many problems and errors. For example, surveying the moose (*Alces alces*) is less exact because their breeding area is much larger [9]. In other cases, not all of the animals can be observed [10], and inferential methods and estimates have been used. In wooded habitats, such as in the case of deer or roe counting, researchers usually work with smaller sample areas, and they interpolate the population data for the entire habitat. Naturally, this does not always lead to accurate estimates [11]. Aerial surveys may still suffer from underestimation due to undetected animals and low precision as a result of inefficient sampling designs [12]. Although the problem of observing animals in wooded areas has not yet been solved, surveying large-bodied animals with aerial methods in sparsely wooded areas is sometimes possible. For example, helicopter-based surveys are efficient at producing population estimates of eastern gray kangaroos (*Macropus giganteus*) in three kangaroo management zones in northeastern New South Wales, Australia [13]. The increased resolution of aerial images increases the recognition accuracy, and it also makes it possible to count the smaller-bodied species. However, counting wild animals remains difficult mainly because even the slightest disturbance causes some species to move [14].

Using analogue film technology, a spatial resolution of approximately 5 cm can be achieved by the economical imaging speed. The resolutions of the digitized high-quality film and digital image (exposed under the same conditions) are the same [15]. Therefore, standard digital CCD and film with similar dynamic and quality attributes produce almost the same image qualities. The quality of images taken by readily available digital CCD and CMOS reached the level of high-quality film technology around 2006. Since then, both top-quality analogue (film recorded) photos and top-quality digital aerial cameras enable high spatial resolution (5–10 cm/pixel) aerial ortho image mapping. While this resolution is appropriate for urban, water and forest cadastral surveys, it is not sufficient for animal counting of smaller species. If the plane flies lower, it can disturb the animals and be dangerous and it violates the rights. In addition, taking images at high speeds at lower altitudes can cause motion blur. However, as noted above, if the ground speed is slower, the animals can move from one flight line to another by the time the aircraft returns on the next line. In the case of short survey lines, increasing the ground speed improves the counting. Some digital aerial cameras are able to take sharp images with a 2.5 cm spatial resolution but without sufficient overlap between subsequent images [16]. The major difficulty occurs when surveying bird populations consisting of medium- and small-sized species. Thus, we developed a new type of aerial camera to address this need, and we created a protocol for its use to alleviate the difficulties of aerial animal counting.

### 2.2. The Common Methods of Wetland Bird Surveying in Hungary

On-site surveys of wetland birds are difficult. Many bird species breed in inaccessible places, for example, in marsh areas. Surveying birds in broad reed beds is a complex task. In the case of passerines, we can estimate the breeding population from the number of singing males in reed beds; however, it is impossible to do an exact survey of herons in this manner. Heron colonies in Central Europe regularly contain several species. The common species can be observed from land when they are flying into the colony. In the case of rare species, we have to spend a long time scanning the sky from a high tower. It is still only possible to approximate the number of wetland birds, especially in mixed and huge colonies. Entering the colony and counting the nests is another method, but orientation in reed beds is difficult and causes disturbance.

Previously used aerial bird surveying methods involved screening small areas and usually involved the use of a low shooting platform [17], which also disturbed the colonies. Manned aircraft and helicopters, as well as UAV, have been used at low altitudes to capture high spatial resolution image data, for example, successful spoonbill surveying with aerial photography. In Hungary, aerial photography surveying has been successfully applied for spoonbill surveying. In the Hortobágy National Park (Central Hungary), aerial surveys have been conducted since 1997 and provide more accurate data than from the ground because colonies are located in dense reed beds [18]. Nests were detectable with aerial surveys, but there was a low detection of medium-sized herons [19]. Unfortunately, classical aerial population estimations, including those of wading birds, introduce errors [20]. These errors may be caused by the distances involved, the disturbance of the birds or inaccurate observations. Small-scale fixed-wing aerial surveys appeared to overestimate the numbers of nesting birds of some species in Louisiana; this bias often increased with the size of the colony [21]. To eliminate this bias, high-resolution image recording procedures are required [22].

To observe individual birds on the ground without disturbing them, an altitude of at least 450 m is stipulated. Thus, the Hungarian 26/2007 (III. 1.) GKM-HM-KvVM joint regulation declares this altitude as the minimal flight altitude to be used above Hungarian national parks [23]. This altitude was also used for lesser flamingo (*Phoenicopterus minor*) when Azure Aerial Photography (Pty) Ltd. conducted an aerial survey at Kamfers Dam in South Africa. The survey was conducted approximately 455 m above the level of the lake [24]. In Hungary, where our sample area is located, previous experimental techniques of waterfowl counting were conducted with gliders and UAVs, but these methods were banned because gliders and UAVs usually fly too low and cause noise.

To successfully count small- and medium-sized species via higher and more unobtrusive surveys, we used a new sensor for the sample area of Lake Velencei (Central Hungary). Our basic endeavor was to reduce the possibility of errors to achieve more accurate results. The most important aspect of the study is to not harm the studied ecosystem. Therefore, a non-disturbing and easily attainable method was developed.

## 3. Material and Method

There is an increasing interest in the possibilities of aerial wildlife surveys and population estimations. The novelty of our method is the combination of high-speed and high-resolution remote sensing and photogrammetry for bird observation with a new high-speed aerial camera, which enables improved spatial resolution, higher flight altitudes, operation in low light conditions and faster surveys. We developed a calibrated digital aerial camera with a rarely achieved high spatial resolution for surveying the nests of waterfowl. The image processing was performed with ortho-rectification after one photogrammetry block triangulation and terrain model extraction [25]. The ortho image mosaic was created by direct orientation and geodetic GCP measurement-based digital ortho-rectification with one photogrammetry block triangulation, beam equalization, digital terrain model extraction, reprojection of images with the DTM and orthophoto mosaicking. The visual interpretation of the generated RGB ortho image mosaic enables us to identify each bird species and to count the number of individual birds.

### 3.1. The Aerial Camera

The quick recorder that was created in the Interspect laboratory by building multiple high-speed camera heads can have an exposure up to 1/8000 s and produce 5–7 shots per second. Its sensitivity and speed enable mapping of the surface in the visible part of the electromagnetic spectrum with a 75° sun angle (measured from the zenith), up to a 0.5 cm spatial resolution without time delayed integration (TDI) or forward motion compensation (FMC). In clear weather conditions, we can produce this resolution at an 800-meter relative altitude. However, in humid weather, it is more practical to achieve the resolution from a lower altitude. The recording is also feasible in low visibility (larger than a 75° sun angle measured from the zenith). Therefore, it is possible to conduct the survey when most of the breeding birds are in their nests. This is a significant advancement considering that certain birds are less active than in the early afternoon. Birds can be identified at the species level even in the images collected under low light conditions. The novelty is that high-quality image recording even becomes possible in overcast weather. The method also works in hazy, rainy weather due to the highly sensitive CMOS available with professional cameras. Table 1 contains the descriptions of the camera. For more details, see [26].

### 3.2. Aircraft Details

During the survey, we used a Piper PA-32 Cherokee Six 300 plane, which was specially modified for remote sensing tasks. This type of plane is equipped with a strong engine, so it can fly continuously and stably even at a 285 km/h ground speed. Two camera systems can be placed face down at the bottom of the plane to obtain simultaneous recording. Additional detectors can be placed into the sidelong window apertures. Furthermore, the electrical system was modified with an auxiliary generator and additional storage batteries; therefore, up to 24 V is available for operation of the instruments.

### 3.3. The Geometric Accuracy of the Aerial Surveys

For the waterland habitat, there was no possibility of measuring the required number of checkpoints to determine the geometric accuracy of the survey. By analyzing the accuracy of our previous surveys and test flights, the expected geometric specifications of the method were determined (Table 2).

**Figure 1.**
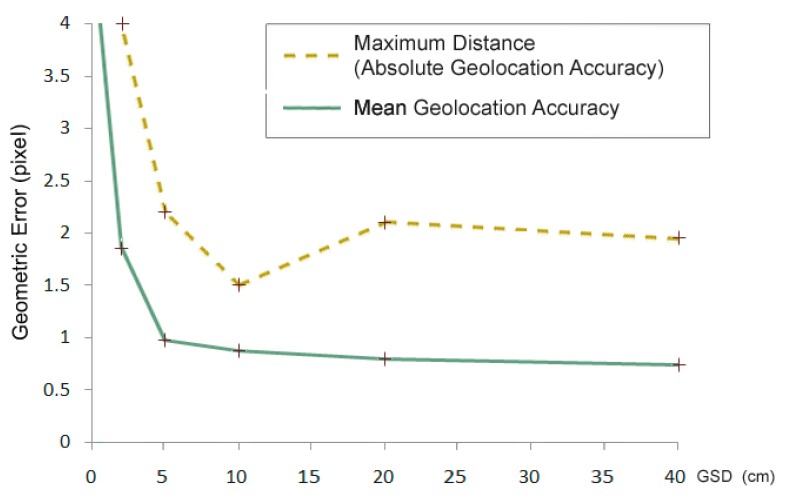
Relative geometric accuracy of different-resolution ortho image mosaics expressed in pixels.

**Figure 2.**
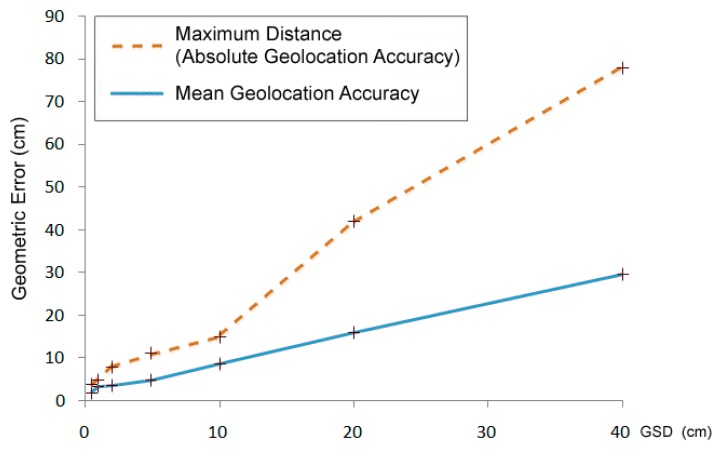
Absolute geometric accuracy of different-resolution ortho image mosaics expressed in cm.

However, by increasing the spatial resolution, the geometric error of elementary pixels also increases (Figure 1), whereas the geographical geometric error decreases (Figure 2). The accuracy of the GPS system of the aerial camera and the precision of the field geodetic GPS systems suitable for checkpoint measurements are a few cm, depending on the surveying operation mode. Therefore, the error measured in the pixels increases in the 1–10 cm range.

In future surveys, the birds and nests on the ground will be displaced from their actual locations on the map with an expected maximum distance of 0.08 m. Neither the 2.19 RMS nor the 8 cm maximum error causes enough local distortion to affect the identification of birds.

### 3.4. The Selection of Sample Areas

**Figure 3.**
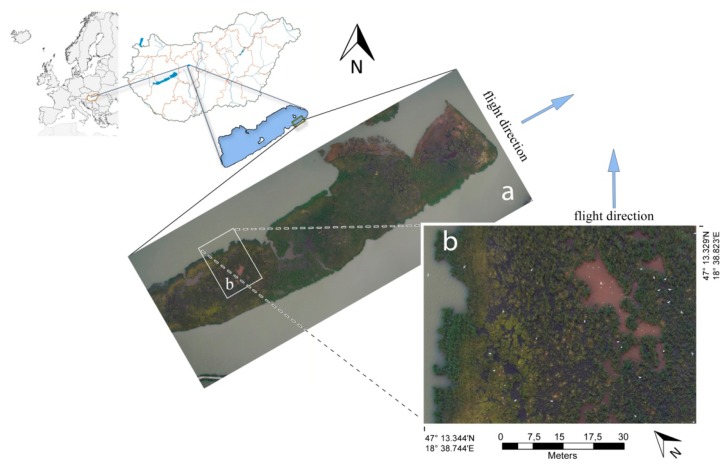
(**a**) Aerial ortho image mosaic of the first flight line of the black-headed gull colony sample area; (**b**) Enlarged detail of Figure 1a.

**Figure 4.**
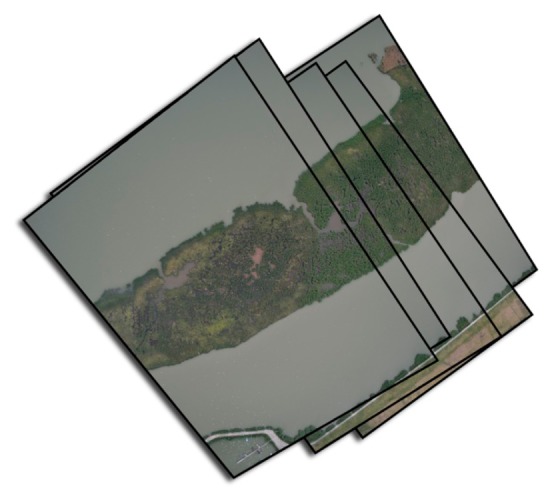
Aerial images of line 1 before photogrammetric processing.

The 280.000 m^2^ research area was located at Lake Velencei where gull colonies (Figure 3) were found in the eastern part of the lake and herons and ducks were found on three islands in the western part of the lake. The area was recorded by two 700 m long flight lines with 37 shots per line over 1.2 min. On average, the recording of one line was 16 s. The aerial survey was conducted on 24 May 2012 from 5:59 to 6:01 p.m. Some images of the first flight line are shown on Figure 4. The experiments comply with the current laws of the country in which they were conducted.

### 3.5. Survey Time

The survey time has to be adjusted to the breeding period of the species. In the case of gulls, the best surveying period is at the end of May or at the beginning of June when black-headed gulls (*Chroicocephalus ridibundus*) and Mediterranean gulls (*Larus melanocephalus*) appear in the colonies. The breeding period of herons is longer than that of gulls. Some species, such as the gray heron (*Ardea cinerea*) or the spoonbill (*Platalea leucorodia*), already appear in breeding places in early spring. Others, such as the glossy ibis (*Plegadis falcinellus*) or the squacco heron (*Ardeola ralloides*), may only start breeding in early summer. The best survey time might be late May when almost every wading bird starts to breed or feed their hatchlings. Before conducting the survey, it is worth inspecting the colonies because the weather often influences the breeding period. Table 3 shows the breeding periods of herons and ibises in Central Europe [27].

### 3.6. The High-Resolution Aerial Survey

The knowledge of habitats helps us classify species by area. Therefore, when using large-scale remote sensing for bird observation, we also apply aerial vegetation mapping to recognize the habitat. Nevertheless, correct identification is based on the detection of the decisive marks of the species and on the resolution of the images.

Based on our experience, it is worth counting waterfowls in the evening hours because, at this time, their movement is less intensive, and they prefer to stay in the colonies. Flying at an appropriately high altitude does not disturb the animals. It is not practical to take highly detailed aerial images from above a 1000 m altitude because the haze between the sensor and the Earth's surface significantly decreases the image quality [28]. We worked at an 800 m relative flight altitude with a 210 m average swath width and an 18–22 m overlap.

**Figure 5.**
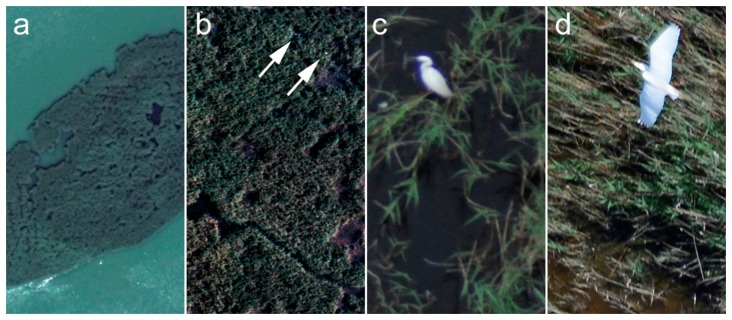
Full resolution details of the sample area: (**a**) satellite image in Google Earth with a 50 cm spatial resolution; (**b**) 10 cm spatial resolution orthophoto section photographed on Kodak film where the arrows indicate two white spots that are possibly flying birds. The 0.5 cm spatial resolution ortho image section recorded by the Interspect calibrated aerial camera of (**c**) a standing heron; and (**d**) a flying heron.

To capture the gull colony, we recorded a 700 m long, 400 m wide area over two flight lines. We strictly monitored the movement of the birds while mosaicking the ortho images line-by-line to avoid the disappearance or reappearance of individual birds on the overlaps. To record the colony, a minimum ground speed of 180 km/h is necessary (even for lines shorter than 700 m) to avoid the movement of the animals between the flight lines. During the 1200-meter long survey of the colonies and habitats, it was found that a 200 km/h ground speed is sufficient to avoid the movement of animals. However, we later chose the more economical 220 km/h speed. Due to the high speed, the telephoto lenses and the low visibility, the images are slightly blurry in full resolution; however, ornithologists are able to confidently identify the species. Figure 5 shows the sharpness of the full resolution images.

In the overlapping parts of the ortho images, the same individual birds are observed. With regard to the full area covered by the image mosaic, there is no risk of double counting at the inline overlaps; however, between two flight lines, a minimal risk is inevitable. The proposed method can be useful in similar conditions with similar or smaller colony sizes, but the success of the general application of the method depends on the movement patterns of the various species and the complexity of their habitats. Software imagery analysis has not yet been developed because, in the overlaps between the lines, the interpreter can usually decide whether each individual bird was identified in the other image sequence.

For larger species, a lower spatial resolution is adequate for identification through wide-angle lenses. Thus, a larger study area can be surveyed. The recognizability of individual birds varies according to species. Our article provides examples of heron, goose, gull and race species to demonstrate the importance of choosing the optimal resolution.

### 3.7. Interpretation at Different Resolutions

We created various spatial resolution ortho images of the sampling area. It was important to use the images from the same flight for recording the individual birds in the same moment and position for the comparison. Thus, the 7, 5, 2.5 and 1 cm ortho image maps were acquired by resampling the 0.5 cm spatial resolution aerial survey. Then, individual birds can be studied at different spatial resolutions in the same conditions. However, the method does not consider the atmospheric effects of the different altitudes. Thus, our conclusions are valid only within the same (ideal) weather conditions. The visual interpretation was initiated with lower spatial resolution images, followed by higher resolution images. The subjective errors were examined at the lowest and highest resolution resampled images by image interpretation involving multiple individuals.

Others have reported the possibilities of mapping birds from aerial image data by automated image analysis procedures. One of these procedures produced counts with a precision comparable to manual counts of the aerial photographs by an expert (<5% difference) [29]. In another experiment, in the object-based image analysis of a 4 cm GSD, the reported overall success level of 18 image frames with respect to under-mapping was over 92% [30]. Therefore, similar experiments should also be conducted in the future with 0.5 cm spatial resolution images.

## 4. Results and Discussion

**Figure 6.**
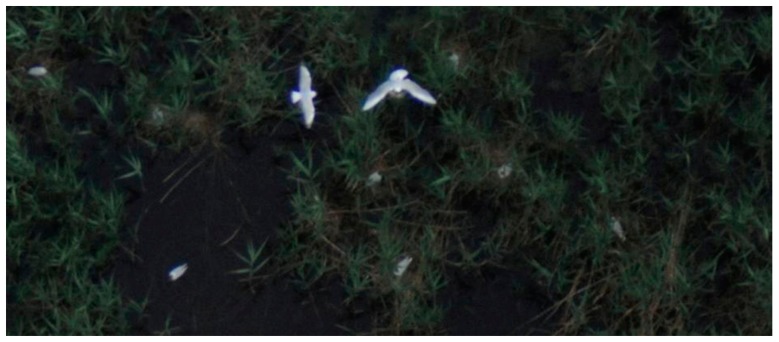
The full resolution shows that stationary and flying birds from the black-headed gull colony are easily identifiable, even in low visibility conditions.

A classic camera cannot capture sharp, bright images in the evening hours when birds are usually in their nests. The new camera system solves this problem. Figure 6 exemplifies an image taken by the IS 3 camera in the evening hours.

The differentiation of bird species in the ortho image map is a completely new challenge, even for skilled ornithologists, because the improved image quality is new. In the 0.5 cm spatial resolution aerial ortho image maps, more bird species are identifiable. We took these images near Lake Velencei where primarily aquatic birds appear.

### 4.1. Bird Species Characteristics from the Air

#### 4.1.1. Great White Egret (*Casmerodius Alba*)

**Figure 7.**
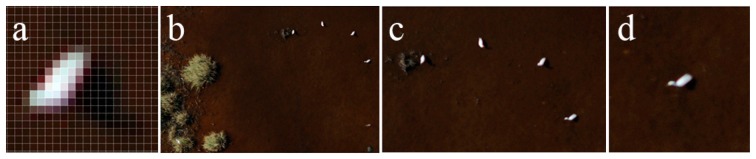
Ortho image map section showing (**a**) a great white egret at the 7 cm spatial resolution (enlarged); (**b**) great white egrets at the 5 cm spatial resolution; (**c**) great white egrets at the 2.5 cm spatial resolution; and (**d**) a great white egret at the 1 cm spatial resolution.

**Figure 8.**
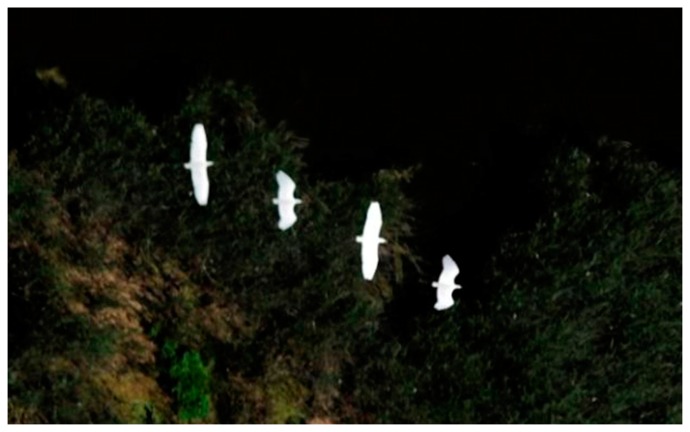
The flight progress of a great white egret in four consecutive images. However, an image is not counted if the plane returns within 0.5 min on the next flight line. The continuous recording also illustrates the flight of the bird.

As a result of the survey, we found that the species cannot be confidently identified in the 7 cm spatial resolution orthophotos. At the 5 cm spatial resolution, the species can be assumed due to its size and white color. Beyond the physical field attributes, the general impression of their size and shape is also useful; thus, the species can also be identified at a lower resolution. At the 2.5 cm spatial resolution, the species is definitely identifiable (the size and color are representative); at the 1 cm spatial resolution, the recognition is unequivocal (Figures 7 and 8). In classic ortho images (50–5 cm/pixel spatial resolution), the species identification is impossible; however, the birds are noticeable in 1 cm or higher spatial resolution ortho images.

The only similar species in the study region is the little egret, which is half the size of the white egret. Their bills are usually yellow; however, at the beginning of the breeding period, their bills are black with a green or pinkish base. Although the bill is not always observed in the images, it can sometimes be difficult to recognize the species. For example, it is difficult to distinguish a flying little egret from a great egret that is standing on the ground. Determining the size of individual birds in flight is difficult if their flight altitude is unknown.

#### 4.1.2. Graylag Goose (*Anser anser*)

**Figure 9.**
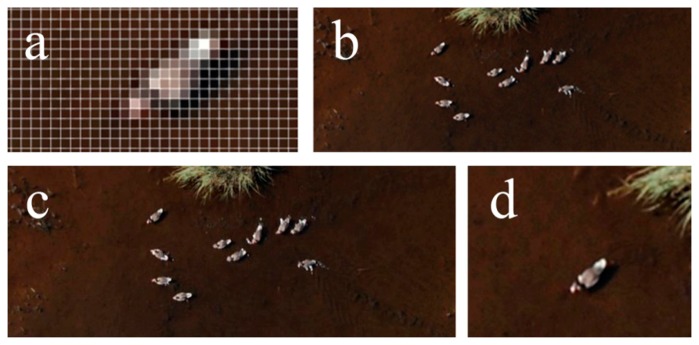
Ortho image map section showing (**a**) a graylag goose at the 7 cm spatial resolution (enlarged); (**b**) graylag geese at the 5 cm spatial resolution; (**c**) graylag geese at the 2.5 cm spatial resolution; (**d**) a graylag goose at the 1 cm spatial resolution.

A graylag goose is easily identified by its huge size, pinkish bill, light-gray upper body and broad white-tipped tail. This species cannot be identified in the 7 cm spatial resolution orthophoto. At the 5 cm spatial resolution, the graylag cannot be confidently differentiated from other goose species. In the 3 cm spatial resolution orthophoto, due to its markings and size, the graylag goose is distinctly identifiable (Figure 9). In classic ortho images, the species is impossible to identify; however, they are noticeable in 1 cm or higher spatial resolution ortho images.

#### 4.1.3. Mallard (*Anas platyrinchos*)

Adult males are identified by breeding plumage, a greenish-yellow bill, a dark glossy green head, a gray back, and a black line down the middle of the upper part of the back. Females and eclipse males are similar to gadwall (*Anas strepera*), but the mallard's tail feathers are almost fully white.

**Figure 10.**
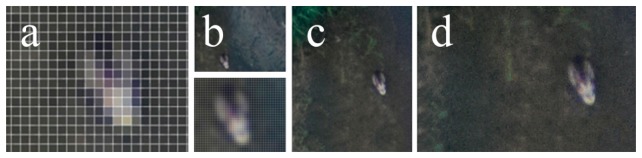
Ortho image map section showing (**a**) a mallard at the 7 cm spatial resolution (enlarged); (**b**) a mallard at the 5 cm spatial resolution; (**c**) a mallard at the 2.5 cm spatial resolution; and (**d**) a mallard at the 1 cm spatial resolution.

The species cannot be identified in the 7 cm spatial resolution orthophoto. At the 5 cm spatial resolution, the bird is unequivocally recognizable due to its shape (it is one of the teal species), but the species can only be identified at the 3 cm spatial resolution when the male's markings are visible. The mallards cannot be identified, even at the 1 cm spatial resolution, by their visual features (Figure 10).

#### 4.1.4. Black-Headed Gull (*Chroicocephalus Ridibundus*)

Adults with breeding plumage have a chocolate brown head, a light gray back and a white tail. Adults with winter plumage have a white head with a black ear spot. This species is easily distinguishable from other common gulls when flying due to the white leading edge of its outer wing. Juveniles have ginger-brown patterns on their backs [31]. The only similar species is the Mediterranean gull, which has a fully white upper body and a blood-red bill.

**Figure 11.**
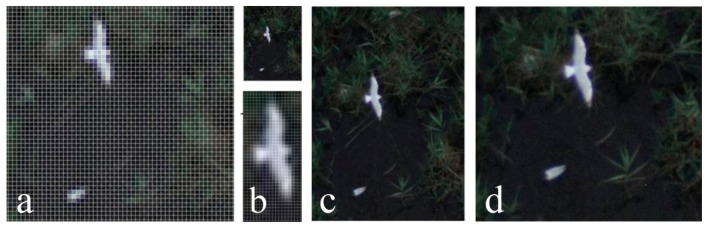
Ortho image map section showing (**a**) black-headed gulls at the 5 cm spatial resolution (enlarged); (**b**) black-headed gulls at the 2.5 cm spatial resolution; (**c**) black-headed gulls at the 1 cm spatial resolution; and (**d**) black-headed gulls at the 0.5 cm spatial resolution.

At the 3 cm spatial resolution, a gull is recognizable regardless of its wings (Figure 11). Nevertheless, the recognition of the species is only possible at the 2 cm spatial resolution because, in this size range, the markings of the wings are also visible. Using classic ortho images, the species cannot be identified; however, they are noticeable in 1 cm or higher spatial resolution ortho images.

### 4.2. Black-Headed Gull Colony in the Orthophoto Mosaic

A black-headed gull colony was previously surveyed at Catalonia (Spain) [32]; the breeding colony was recorded by micro UAS technology. Our images obtained by IS 3 differ from this previous study in terms of the surveying speed and distance. The UAS speed was 30–40 km/h at 30–40 m above the ground, while our flight was 220 km/h at 500 m above the ground. The spatial resolution of our survey is equivalent to or higher than the cited UAS survey.

Our ortho images of black-headed gull breeding colonies in a reed island near the town of Velence were evaluated by visual interpreters, including professional fowlers. Point vector GIS overlays of the breeding colonies were performed with Global Mapper 2.14 software.

**Figure 12.**
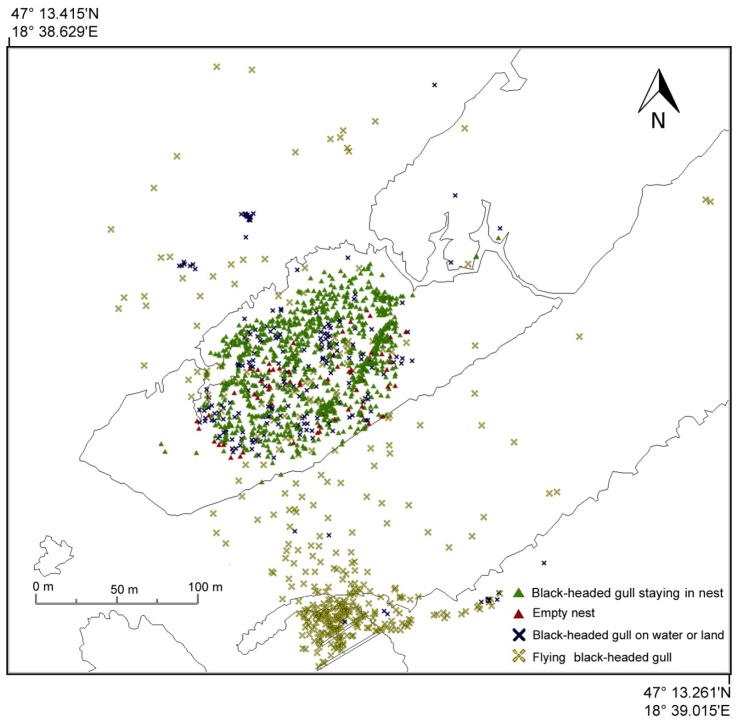
Evaluated black-headed gull colony.

Separate point vector overlays were created for individual black-headed gulls that flew above or close to the colony; another overlay was created for black-headed gulls that stayed at the nest. A vector layer represents the black-headed gulls on the water or the island, and another layer represents the empty nests. These point vectors represent the gulls and the nests at the time of the aerial survey (Figure 12).

At the time of the survey, 836 black-headed gulls were in nests, 220 were on the water or on the ground, 59 nests were empty, and 374 individual birds were flying within the 182,000 m^2^ sample area of the reed island. Figure 1 illustrates the flight scan, while Figure 7 represents the interpretation of the derived map of the colony.

### 4.3. The Reliability of the Evaluation

Although the test flight could not be repeated during the same breeding period as the imaging, the reliability of the visual interpretation was verified by the evaluation of the images by five independent persons. The validation of the method was conducted at the Budapest Zoo and Botanical Garden under verifiable conditions.

#### 4.3.1. Examining the Subjective Errors

With regard to the number of individual birds on the water, on the ground and in flight, the results of the five interpreters did not differ. Regarding black-headed gulls in the nest, one of the interpreters counted one less individual; consequently, the evaluation is considered to have a reliability level of 99.93%. The visibility of empty nests was significantly worse than the visibility of birds. Thus, the level of accuracy for the detection of empty nests was only 68% for the ortho image with a 0.5 cm ground sample distance (GSD). For the 7 cm spatial resolution, the evaluation was unequivocal only for flying birds. Furthermore, the reduced (7 cm GSD resampled) ortho image map was analyzed by multiple persons. Table 4 shows the evaluation of the five interpreters. If we accept the results of the survey with a 0.5 GSD, then the average deviation and the maximum error of the data derived from evaluating the orthophoto of 7 cm GSD can be calculated relative to the acceptable results. The data are shown in the last two columns of the table.

#### 4.3.2. Field Validation of the Aerial Survey

A count of the birds on the reed island and the birds in flight was attempted from a boat only minutes after the flight, but accessing the area and the orientation proved to be difficult. Due to the large number of individual birds and the limited visibility, the field validation of the aerial survey was not possible in the natural habitat.

**Figure 13.**
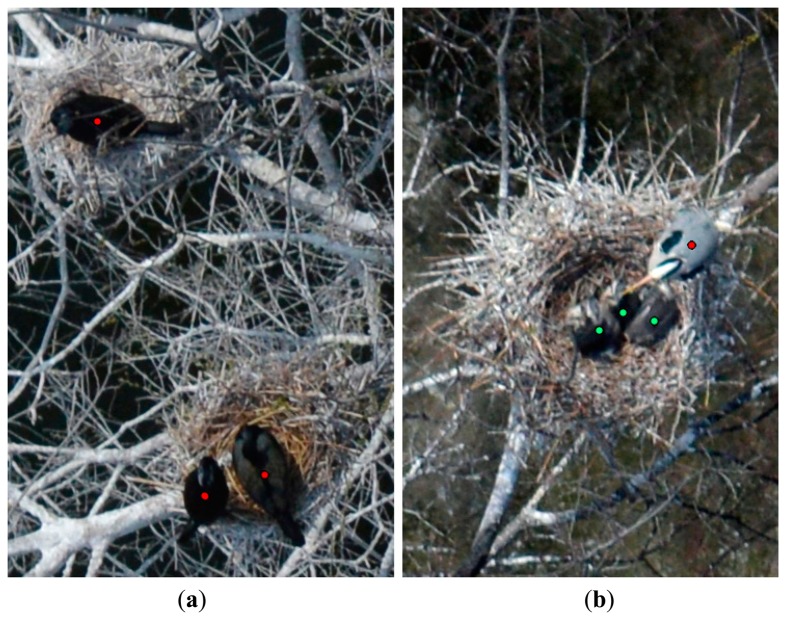
(**a**) Great cormorants and (**b**) gray heron nest with three hatchlings and an adult at the top of a tree in the Budapest Zoo & Botanical Garden. (Adults are marked with red points, and hatchlings are marked with green points).

The experiment was repeated under controlled conditions with the ornithologists of the Budapest Zoo & Botanical Garden. Over select areas of the zoo where trees do not cover the animals, the aerial survey was conducted. Meanwhile, the zoologists verified the number of animals in the selected enclosures. Comparing the number of identified birds in the ortho image mosaic with the data in the report that records the number of field-observed bird individuals at the time of the overflight, it was found that the aerial colony-size monitoring of the species in Table 5 led to accurate results (for the high spatial resolution true color aerial survey in an uncovered area). Figure 13 displays ortho image mosaic sections of the Budapest Zoo & Botanical Garden.

## 5. Conclusions

Using the camera system developed by the Interspect Research Group, we were able to survey bird colonies from aircraft at a relatively high altitude; the captured images were processed by photogrammetric methods. The quality and spatial resolution of the images permitted the identification of species and counts of nests in the vicinity. In some cases, it was possible to determine the number of hatchlings in the nests. The issue of surveying habitats covered with trees and high vegetation remains to be solved. The greatest advantage of aerial observation is that it does not disturb the animals. On-site observations that last for days can be substituted in many cases. If on-site visits are needed for some reason, the exact locations of the nests are known thanks to GPS coordinates; thus, the disturbance of intervention can be minimized. A further advantage of ortho image maps is that analyses of the colony are possible at a later stage; therefore, contingent errors can be revised. Often, it is difficult to distinguish the birds through on-site observation, especially in low-light conditions from far distances because an individual bird can sometimes only be observed for a moment. In this case, even an experienced ornithologist might mistake one species for another similar species. By using the proposed method, we can determine how many individual birds stayed in the surveyed area during the surveying period while also considering underestimation or overestimation. Several surveys should be conducted during the breeding period because a single survey does not guarantee that all birds are in the colony at the same time. It is worth repeating the surveys on at least three consecutive days to obtain more accurate data on the number of birds in the colony.

It was observed that higher spatial resolutions result in visible shadows in more pixels due to the high level of detail. Therefore, the hue range and the dynamic range enable us to recognize the animals, even in shaded areas.

## Figures and Tables

**Table 1.** The test-relevant data of the IS 3 test frame camera system.

**Type**	**INTERSPECT IS 3 MS**
Sensor	RGB: CMOS in modular configuration
Sensor size	36 × 24 mm
Resolution of the camera parts	6286 × 4426 pixels
Pixel size	6 μm
Spatial resolution	100–0.5 cm
Spectral resolution	RGB, UV, NIR 356 nm–1180 nm

**Table 2.** Accuracy of previous surveys at nature conservation areas (open grasslands).

**GSD**	**Map Scale**	**Max. Distance (cm)**	**Mean (pixel)**	**Mean (cm)**	**Stdev. (pixel)**	**RMSE (pixel)**	**Number of Checkpoints**
40	1:4800	78	0.74	29.67	0.53	0.89	16
20	1:2400	42	0.80	16.00	0.60	0.98	16
10	1:1200	15	0.88	8.78	0.39	0.95	16
5	1:600	11	0.98	4.93	0.6	1.15	15
2	1:240	8	1.87	3.73	1.19	2.19	15
1	1:120	5	3.46	3.46	1.41	3.72	15
0.5	1:60	4	4.13	2.07	2.20	4.65	15

**Table 3.** Breeding periods of herons and ibises in Central Europe.

**Species**	**Breeding Period**
Black-crowned night heron	April–July
Squacco heron	May–July
Little egret	May–August
Great egret	April–July
Gray heron	April–July
Purple heron	April–July
Glossy ibis	May–July
Spoonbill	March–July

**Table 4.** Interpreter results from the 7 cm spatial resolution ortho images.

	**Results of Interpreters**							**Compared with the 0.5 cm GSD Survey**	

	**1**	**2**	**3**	**4**	**5**	**Mean**	**Stdev.**	**Average Error**	**Maximum Error**
On water or land	417	416	433	402	385	410.60	18.04	190.6	213
In the nests	581	644	527	652	558	592.40	54.33	243.6	309
Flying gulls	374	374	374	374	374	374.00	0.00	0	0
Empty nests	71	27	61	67	49	55.00	17.72	12.72	32
Number of gulls	1372	1434	1334	1428	1317	1377.00	53.21	54.6	113

**Table 5.** Number of individuals in the enclosures and in the foliage that were selected for testing by the Budapest Zoo & Botanical Garden at the time of the overflight based on the field and aerial survey data.

**Species**	**Field Observation**	**Aerial Survey**
White Stork (*Ciconia ciconia*)	8	8
Graylag Goose (*Anser anser*)	4	4
Great Egret (*Egretta Alba*)	3	3
Gray Heron (*Ardea cinerea*)	49	49
Great Cormorant (*Phalacrocorax carbo*)	95	95
Mallard (*Anas platyrinchos*)	48	47
Black-headed Gull (*Chroicocephalus ridibundus*)	8	8
